# Eating away from home among hypertensive patients in Addis Ababa: frequency and association with blood pressure control

**DOI:** 10.1186/s12889-026-26871-x

**Published:** 2026-03-21

**Authors:** Hana Shumetie, Robel Yirgu, Samson Gebremedhin

**Affiliations:** College of Health Sciences, School of Public Health, Addis Ababa University, Addis Ababa, Ethiopia

**Keywords:** Hypertension, Eating away from home, Blood pressure control, Dietary habits, Non-communicable diseases

## Abstract

**Background:**

Eating away from home makes healthy dietary choices challenging. Foods prepared away from home are commonly energy dense, micronutrient poor, and contain high salt making them less preferred from non-communicable disease (NCD)-perspectives and may increase the risk of poor blood pressure control. In the Ethiopian context, the frequency of eating away from home and its association with blood pressure control have not been explored before.

**Objectives:**

To assess the frequency of consumption of meals prepared away from home among hypertension patients in Addis Ababa, Ethiopia, and determine its association with blood pressure control.

**Methods:**

An institutional-based cross-sectional study was conducted from January to March 2025 among 474 hypertensive patients randomly selected from public hospitals and health centers in Addis Ababa. Frequency of eating away from home (EAFH) was assessed using a standard questionnaire and categorized as low (< 3 times/week) or high (≥ 3 times/week). Blood pressure (BP) was measured using validated digital sphygmomanometers and poor blood pressure control was defined as systolic BP ≥ 130 mm Hg or diastolic BP ≥ 80 mm Hg. Binary logistic regression analysis was used to evaluate the association between EAFH frequency and blood pressure control, adjusting for socio demographic, clinical and behavioral factors.

**Results:**

A substantial burden of poor blood pressure control was observed among the study participants. The prevalence of uncontrolled blood pressure among the participants was 68.9% (95% CI: 64.7% -73.1%). About 24.6% of participants had high eating away from home (3 or more times per week). High frequency of EAFH was significantly associated with increased odds of uncontrolled blood pressure (AOR = 1.77; 95% CI: 1.04-3.00; COR = 2.01; 95% CI: 1.31–3.11).

**Conclusion:**

Frequent consumption of meals prepared away from home is significantly associated with poor blood pressure control among hypertensive patients. Public health interventions should focus on raising awareness about the health implications of EAFH, promote healthier food environments, and support hypertensive individuals in making better dietary choices.

**Supplementary Information:**

The online version contains supplementary material available at 10.1186/s12889-026-26871-x.

## Background

Eating away from home conventionally refers to food items that are obtained, although not exclusively, from restaurants, cafeterias, food trucks, street foods, and other commercial or noncommercial food service establishments [1]. Eating out is frequently linked to unhealthy eating habits and increased blood pressure [2, 3]. Individuals who eat out often tend to consume fewer essential nutrients, such as dietary fibers, vitamins, and minerals like potassium and magnesium, which are important in managing hypertension [4]. Meals prepared outside the home usually contain higher sodium levels due to excess use of salt as a flavoring agent and a preservative [5]. This excess sodium contributes to hypertension and other cardiovascular problems [6, 7].

Most of the time, when people eat outside their home, the meals they choose are influenced by recognizable brands that they have seen through marketing, encouraging them to consume these brands frequently [8]. Consumption of unhealthy foods in social gatherings is also one way individuals consume calorie-dense foods (foods high in energy, typically from fats and/or added sugars). Events, peer influence, and social media advocate unhealthy eating practices such as overeating [9]. Appealing pictures and marketing of these fast- foods also made especially children to have positive perception on eating unhealthy foods which will eventually lead them to long term health complications [10].

Globally, more than 1.2 billion adults are affected by hypertension and the highest prevalence is reported in adults 30 and 79 years of age. Out of the affected population, two-thirds live in low and middle income countries (LMICs) [11]. The condition is also a major concern in Sub-Saharan Africa (SSA), affecting 30.8% of the population [12]. In Ethiopia, the estimated hypertension prevalence ranges, from 15% to 30% [13]. According to a systematic review conducted in Ethiopia, the total prevalence of hypertension was 19.6%, with a higher prevalence in urban areas (23.5%) than in rural areas (14.7%). The same study also reported that the prevalence of hypertension was slightly higher among males (20.6%) than females (19.2%) [14].

Uncontrolled hypertension, defined as a blood pressure measurement with a systolic pressure of ≥ 130 mm Hg or a diastolic pressure of ≥ 80 mm Hg [15], imposes substantial health and financial burdens, including disability-adjusted life years (DALYs). Many studies in Ethiopia and beyond indicated a substantial proportion of hypertensive patients on treatment fail to control their blood pressure. The Prospective Urban Rural Epidemiology (PURE) study indicated only 32.5% of treated patients in different countries achieved sufficient control [16]. In Kenya, only 33.4% of hypertensive patients maintained recommended blood pressure levels [17]. A study in Brazilian clinic found nearly half of patients did not achieve controlled blood pressure [18]. A systematic review in Ethiopia found about 51% of hypertensive patients had uncontrolled blood pressure, with Addis Ababa’s prevalence higher at 58% [19]. Factors contributing to this poor blood pressure control include provider-related issues like neglecting treatment, irregular follow-up scheduling, and lack of awareness about the treatment [20], while patient factors include low medication adherence, poor lifestyle choices, and chronic conditions [21].

As urbanization expands, individuals have increased consuming meals outside their home that worsens hypertension [22]. This study examining the relationship between eating out frequency and blood pressure control among hypertensive patients in Addis Ababa provides valuable information for healthcare professionals, policy makers, patients and the community at large. Even though prior studies have showed that there is strong connection between cardiovascular health and dietary patterns [23], this study has delved into hypertensive patients’ dietary habits in order to understand how frequency of eating out influences blood pressure control. The purpose of the study was to assess the frequency of consumption of food prepared away from home among hypertensive patients in Addis Ababa and to determine its association with blood pressure control.

## Methods and materials

### Study design, period, source and study population

This institution-based cross-sectional quantitative study was conducted from January to March 2025 in Addis Ababa, Ethiopia. Data was collected from selected public hospitals and health centers, including Eka Kotebe General Hospital, Tirunesh Beijing General Hospital, Yekatit 12 Hospital, and six health centers: Meshualekia, Amoraw, Felege Hiwot, Kotebe, Teklehaimanot, and Saris. The study population included all hypertensive patients above 18 years residing in Addis Ababa who were on medical follow-up at these facilities during the study period. Participants were those with at least one prior follow-up visit to ensure availability of clinical history for accurate characterization of hypertension management. Critically ill hypertensive patients, pregnant women, and nursing mothers were excluded to avoid potential confounding from physiological changes during pregnancy and lactation that could independently influence blood pressure control and dietary behaviors.

### Sample size and sampling procedure

The sample size was calculated using a 95% confidence level, 24.4% anticipated prevalence from a previous study in Hawassa [24], 5% margin of error, 10% for possible non-response, and a design effect of 1.5 for the multistage sampling approach. The ultimate sample size was 474. The sample size was allocated proportionally to each selected facility based on the number of patient flow. A multi-stage stratified sampling technique was used to select study participants. Three public hospitals were selected from twelve using simple random sampling, while six health centers were selected from ninety-eight using systematic random sampling with a sampling interval of sixteen. Systematic random sampling was then applied to select participants from each facility. Patients arriving at the clinics for their follow-up visits were interviewed. Follow-up visits are appointment-based, allowing systematic random selection.

### Data collection: tools and procedures

Data was collected from January to March 2025 by interviewing respondents using a structured questionnaire. Before data collection, a pretest was conducted on hypertensive patients from Hidasse Health Center, which was not included in the study. Four nurses, trained for 2 days on the study’s purpose, methods and use of the Kobo Toolbox app, collected data during scheduled follow-up appointments from Monday to Friday. Blood pressure and anthropometric measurements were taken after the interview.

The Kobo Toolbox app was used to collect data on demographic and socio-economic information, clinical characteristics, behavioral and dietary factors, EAFH, and measurements. Demographic data included sex, age, marital status, education, occupation, and monthly income. Clinical characteristics assessed hypertension diagnosis, comorbidities, complications and medication use, with questions adapted from World Health Organization (WHO) STEPwise Approach to Surveillance (STEPS) instruments [25].

Behavioral factors included physical activity (assessed by WHO Global Physical Activity Questionnaire), tobacco and alcohol use (STEPS questions), and khat use (standardized questionnaire) [26]. Dietary factors focused on salt intake and fruit and vegetable consumption, based on WHO STEPS dietary modules.

Height and weight were measured to calculate Body Mass Index (BMI) using the formula: BMI = weight (kg) / height (m²). Blood pressure control was coded as binary: 1 for uncontrolled hypertension (Systolic blood pressure (SBP) ≥ 130 mm Hg or Diastolic blood pressure (DBP) ≥ 80 mm Hg) and 0 for controlled hypertension, following American College of Cardiology (ACC) and American Heart Association (AHA) guidelines [15].

Blood pressure was measured with clinically validated Yuwell digital sphygmomanometers, following WHO guidelines. Participants rested seated for 5 min before two readings were taken 1–2 min apart and the average was recorded. Physical activity was self-reported based on engagement in moderate and vigorous activities during work, transport, and leisure, as well as time spent sitting, walking, or cycling.

Eating away from home was assessed using a standardized questionnaire [27]. Participants reported the frequency, type, and context of meals eaten outside over the past 7 and 30 days, including fast food, sit-down restaurants, buffets, takeaway, and street foods (e.g., injera, kokor, pasti, bonbolino), along with reasons for EAFH, knowledge and attitude of the participants on EAFH.

The questionnaire used in this study was developed by the investigators based on standard instruments, primarily the WHO STEPwise Approach to Surveillance (STEPS) questionnaire and the Global Physical Activity Questionnaire, with minor adaptations to the local context. The English version of the questionnaire is provided as a supplementary file.

### Data management and analysis

The collected data was exported from the Kobo Toolbox app and imported into SPSS for analysis. The data was examined for outliers and adjusted as necessary. Multiple related variables were combined into indices or categorized variables, including total comorbidity, clinical characteristics, physical activity, tobacco and alcohol use, khat consumption, EAFH frequency, and BMI, based on standard guidelines and prior literature. Descriptive statistics was used to summarize demographic and socio-economic variables, clinical characteristics, behavioral factors, eating habits, and blood pressure level. Logistic regression analysis was used to assess the relationship between EAFH frequency and blood pressure control. Candidate variables for adjustment in the multivariable model were selected based on theoretical relevance, previous literature, and statistical criteria from the bivariate analysis. These included socio-demographic characteristics (age, sex, marital status, education, occupation, and income), clinical indicators (duration of hypertension and clinical characteristics), and behavioral factors (physical activity and substance use like alcohol, tobacco, and khat). Dietary factors such as salt intake, fruit and vegetable consumption, and BMI were excluded as potential mediators or confounders. Potential confounders were identified through literature reviews. Odds ratios and 95% confidence intervals were identified for each confounder. After selection, variables were incorporated into the logistic regression model as covariates. Independence of observations, outliers, and multicollinearity were checked. The model showed moderate predictive performance with an overall classification accuracy of 73.9%. It effectively identified uncontrolled blood pressure cases (91.8%; 291/317) but was less effective for controlled cases (34.3%; 49/143), indicating stronger sensitivity for high-risk individuals. The model was statistically significant (Omnibus Test χ² = 67.557, df = 42, *p* = 0.007), confirming at least one meaningful predictor. Explanatory power was moderate (Cox & Snell R² = 0.137; Nagelkerke R² = 0.192). The Hosmer and Lemeshow Test (χ² = 4.271, df = 8, *p* = 0.832) indicated good model fit.

## Results

### Demographic and socio-economic characteristics

The study included 460 hypertensive patients from nine health institutions (3 public hospitals and 6 health centers), yielding a 97% response rate. 60% came from health centers, 40% from public hospitals. The male-to-female ratio was 1.12. Participants’ mean age was 50.6 (± 11.1) years, with 73.5% aged over 45 years. 54.3% were married, and 49.6% attained tertiary-level education. Nearly half of them (45.4%) held professional/technical/managerial jobs. Median monthly household income was 15,000 ETB (Table 1).

**Table 1:** Demographic and socio-economic characteristics of hypertensive patients in Addis Ababa, January–March 2025

Variables	Frequency (*n* = 460)	Percent
Sex		
Male	243	52.8
Female	217	47.2
Age in years		
25–34	37	8.0
35–44	85	18.5
45–54	163	35.4
55–64	70	25.9
Above 65	56	12.2
Marital status		
Currently married	250	54.3
Never married	61	13.3
Divorced	59	12.8
Widowed	55	12.0
Separated	35	7.6
Level of education		
Can’t read and write	18	3.9
Can read and write	43	9.3
Primary education (1–4)	16	3.5
Primary education (5–8)	40	8.7
Secondary education (9–12)	115	25.0
Tertiary education	228	49.6
Occupation		
Professional/technical/managerial	209	45.4
Sales and services	74	16.1
Unemployed/Jobless	67	14.6
Skilled manual	50	10.9
Unskilled manual	34	7.4
Domestic service	26	5.7
Household income (in ETB)		
Less than 10,000	151	32.8
10,000–19,999	235	51.1
20,000–29,999	49	10.7
30,000–39,999	17	3.7
40,000 or more	8	1.7
Personal income (in ETB)		
Less than 5,000	79	17.2
5,000–9,999	268	58.3
10,000–19,999	81	17.6
20,000–29,999	23	5.0
30,000–39,999	9	2.0

### Clinical characteristics

More than half were diagnosed with hypertension one year prior. 35% had no comorbidities, while 6.7% had obesity and 4.6% had diabetes. Stroke (23%) was the most common complication, followed by heart disease (16.1%) and chronic kidney disease (10.2%). Most of the study subjects (78.3%) were currently on antihypertensive medication, while 17.6% had previously taken it but were not currently taking it. 56.7% monitored their BP at least twice weekly. Follow-up schedules were mostly monthly (28.9%) or quarterly (24.8%), though 18.3% had provider-scheduled visits (Table 2).

**Table 2:** Clinical characteristics of hypertensive patients in Addis Ababa, January–March 2025

Variables	Frequency (*n* = 460)	Percent
Time since hypertension diagnosis		
Within the last 6 months	105	22.8
6 months to 1 year ago	98	21.3
1 to 3 years ago	95	20.7
More than 3 years ago	118	25.7
I don’t remember	44	9.6
Comorbid conditions associated with hypertension*		
No comorbidity	161	35.0
Obesity	31	6.7
Diabetes	21	4.6
Complications*		
Stroke	106	23.0
Heart disease	74	16.1
Chronic kidney disease	47	10.2
Medications use for other chronic condition		
Yes	242	52.6
No	218	47.4
Medication use history (*n* = 460)		
Currently on HTN medication	209	45.4
Previously on HTN medication	74	16.1
Prescribed medication but not started	50	10.9
Not prescribed any HTN medication	34	7.4
Blood pressure monitoring at least twice a week		
Yes	261	56.7
No	199	43.3
Follow up frequency (*n* = 460)		
Weekly	90	19.6
Monthly	133	28.9
Every 3 months	114	24.8
Every 6 months	36	7.8
Annually	3	0.7
As scheduled by the healthcare provider	64	18.3

*- Multiple response possible

### Level of physical activity

Most participants (51.3%) had a high total physical activity level. Only 12.4% performed vigorous-intensity activity and 25% of them performed moderate exercise. Time spent in work, recreational, and transport physical activities was low, with 89.8% and 89.6% not doing vigorous work and vigorous recreational activity, respectively. Sedentary behavior was notable, with 46.1% reporting high sedentary time.

### Substance use history

Tobacco use was low, with 92% never using it and 1.7% daily smokers. Alcohol consumption was more prevalent, with 30.4% reporting use in the past 12 months and 27.8% in the last month, including 8.7% binge drinkers and 3% high-intensity drinkers. Khat use was reported by 19.6%, with 8.9% chewing daily (Table 3).

**Table 3:** Substance use among hypertensive patients in Addis Ababa, January–March 2025

Variables	Frequency (*n* = 460)	Percent
Current tobacco use		
Yes	37	8.0
No	423	92.0
Daily tobacco use		
Doesn’t smoke	423	92.0
Yes	29	6.3
No	8	1.7
Years of smoking		
Doesn’t smoke	423	92.0
Short term smoking	4	0.9
Long term smoking	33	7.2
Type of smoke		
Doesn’t smoke	423	92.0
Manufactured cigarettes	32	7.0
Hand-rolled cigarettes	3	0.7
Cigars, cheroots, cigarillos	2	0.4
Ever consumed alcohol		
Yes	143	31.1
No	317	68.9
Past 12 months alcohol consumption		
Doesn’t consume	317	68.9
Yes	140	30.4
No	3	0.7
Number of occasions of alcohol consumption in the past 12 months		
Doesn’t consume	317	68.9
Daily	23	5.0
5–6 days per week	7	1.5
1–4 days per week	41	8.9
1–3 days per month	45	9.8
Less than once a month	27	5.9
Alcohol consumption in the past 30 days		
Doesn’t consume	317	68.9
Yes	128	27.8
No	15	3.3
Average standard drinks per occasion in the past 30 days		
Doesn’t consume	328	71.3
Infrequent	62	13.5
Occasional	46	10.0
Frequent	24	5.2
One occasion Standard drinks		
Doesn’t consume	328	71.3
Low risk consumption	62	13.5
Moderate risk consumption	46	10.0
High risk consumption	24	5.2
Largest number of drinks on a single occasion in the past 30 days		
Doesn’t consume	328	71.1
Low consumption	79	17.1
Binge drinking	40	8.7
High-intensity drinking	14	3.0
Khat use		
Yes	90	19.6
No	370	80.4
Frequency of khat use		
Doesn’t consume	370	80.4
Daily	41	8.9
Three times a week	26	5.7
Once a week	16	3.5
Once a month	7	1.5
Amount of khat chewed per session		
Doesn’t consume	370	80.4
Mild	39	8.5
Moderate	42	9.1
Heavy	7	1.5
Don’t remember	2	0.4

### Prevalence of overweight and obesity

In terms of BMI, a substantial portion were either overweight (31%) or obese (12%), indicating a considerable burden of excess weight among hypertensive patients.

### Dietary habits

Dietary patterns showed significant variations in salt use and produce intake. Most of them actively limited salt, with 38.3% reducing salt during cooking. Fruit and vegetable consumption was low. 17.8% and 11.3% ate no fruit and vegetables in the past week respectively. Only 3.7% and 10.2% consumed daily fruits and vegetables. By servings, 55.4% had very low fruit intake (< 2 servings/day). Only 3.5% met WHO recommendations (≥ 5 servings/day) (Table 4).

**Table 4:** Dietary pattern of hypertensive patients in Addis Ababa, January–March 2025

Variables	Frequency (*n* = 460)	Percent
Limit salt intake in any way		
Yes	321	69.8
No	139	30.2
Methods used to reduce salt in diet*		
Avoid processed foods	207	45.0
Add less salt during cooking	176	38.3
Avoid salty snacks	158	34.3
Fruit consumption days per week		
No consumption	82	17.8
Minimal consumption	153	33.3
Moderate consumption	138	30.0
Frequent consumption	70	15.2
Daily consumption	17	3.7
Vegetable consumption days per week		
No consumption	52	11.3
Minimal consumption	155	9.3
Moderate consumption	139	3.5
Frequent consumption	67	8.7
Daily consumption	47	25.0
Fruit servings per occasion		
Very low intake	255	55.4
Low intake	170	37.0
Moderate intake	30	6.5
High intake	5	1.1
Vegetable servings per occasion		
Very low intake	75	16.3
Low intake	195	42.4
Moderate intake	137	29.8
High intake	53	11.5

*- Multiple response possible

### Eating away from home

Eating away from home refers to consuming foods or beverages prepared outside ones’ household, including restaurant meals, fast food, street vendors, cafeterias, and takeaway or delivery foods. For this study a composite EAFH index was created from nine questions about frequency, type, and reasons for eating out. Participants were categorized as low EAFH (< 3 times/week) or high EAFH (≥ 3 times/week). Participants who never ate away from home were included in the low EAFH category. Results showed 24.6% had high EAFH frequency (Table 5).

**Table 5:** Frequency of eating away from home by food type among hypertensive patients in Addis Ababa, January–March 2025

Frequency of eating away from home (*n* = 460)									
Type of Food/Setting	Never/Rarely	1×/Month	2–3×/Month	1–2×/Week	3–4×/Week	5–6×/Week	1×/Day	2×/Day	≥ 3×/Day
Fast food restaurant	90 (19.6%)	48 (10.4%)	40 (8.7%)	51 (11.1%)	25 (5.4%)	35 (7.6%)	80 (17.4%)	25 (5.4%)	66 (14.3%)
Sit-down restaurant	92 (20.0%)	49 (10.7%)	41 (8.9%)	52 (11.3%)	26 (5.7%)	36 (7.8%)	81 (17.6%)	26 (5.7%)	67 (14.6%)
Buffet meals	250 (54.3%)	46 (10.0%)	35 (7.6%)	35 (7.6%)	22 (4.8%)	34 (7.4%)	23 (5.0%)	7 (1.5%)	8 (1.7%)
Sweet foods (prepared outside)	265 (57.6%)	51 (11.1%)	35 (7.6%)	45 (9.8%)	18 (3.9%)	20 (4.3%)	20 (4.3%)	5 (1.1%)	1 (0.2%)
Takeaway food	252 (54.8%)	51 (11.1%)	39 (8.5%)	40 (8.7%)	25 (5.4%)	10 (2.2%)	28 (6.1%)	8 (1.7%)	7 (1.5%)

Among those who ate away from home, the most commonly consumed food types were street foods such as Injera, Kokor, and Ertib (62.4%), followed by cakes and bakery items (42.4%) and fast foods like burgers and fries (29.6%). The main reasons cited for eating out included taste preference (57.2%), convenience (50.4%), and socializing (43.9%) (Table 6).

**Table 6:** Types of meals consumed outside the home and motivational factors among hypertensive patients in Addis Ababa, January –March 2025

Variables	Frequency (*n* = 460)	Percent
Eating meals prepared away from home number in the past week		
Never or rarely	257	55.9
Occasionally	90	19.6
Frequently	75	16.3
Very Frequently	38	8.3
Types of takeaway meals frequency*		
Fast food such as Burgers, fries, chicken nuggets, pizza	93	20.2
Casual dining meals like international cuisines	125	27.2
Street foods like Injera, kokor or pasti, bonbolino and ertib	168	36.5
Cakes or bakeries	292	62.8
Sweets and snacks	92	20
Types of meals while eating away from home*		
Fast food such as Burgers, fries, chicken nuggets, pizza	136	29.6
Casual dining meals like international cuisines	41	8.9
Street foods like Injera, kokor or pasti, bonbolino and ertib	287	62.4
Cakes or bakeries	195	42.4
Sweets and snacks	113	24.6
Reasons to eat away from home*		
Convenience	232	50.4
For socializing purpose	202	43.9
Peer influence	111	24.1
For luxury	120	26.1
Lack of cooking skills	72	15.7
Social media marketing	54	11.7
Taste preference	263	57.2
Busy lifestyle	162	35.2

* - Multiple response possible

### Prevalence of uncontrolled hypertension

A majority of participants (68.9%, 95% CI: 64.7–73.1%) had uncontrolled blood pressure, with similar prevalence in males (64.6%) and females (73.7%). By age, the highest prevalence was in 45–54 years (25.0%), followed by 55–64 years (17.6%) and 35–44 years (13.0%).

### Association between frequency of eating away from home and blood pressure control

Binary logistic regression analyzed EAFH frequency and blood pressure control in Addis Ababa hypertensive patients, adjusting for age, sex, BMI, income, physical activity, smoking, alcohol, khat use, and comorbidities. Blood pressure control was binary (0 = controlled, 1 = uncontrolled). Frequent EAFH consumers had 2.02 times higher odds of uncontrolled blood pressure versus infrequent consumers (COR = 2.02; 95% CI:1.31–3.11; AOR = 1.77; 95% CI :1.04-3.00), showing a strong association between frequent eating out and poorer BP control (Table 7).

**Table 7:** Crude and Adjusted Odds Ratios for the association between frequency of eating away from home and blood pressure control in Addis Ababa, January–March 2025

Variables	Blood pressure control		COR (95% CI)	AOR (95%CI)
	Uncontrolled (*n*, %)	Controlled(*n*, %)		
EAFH frequency				
Low	70 (57.4%)	52 (42.6%)	1.00	1.00
High	247 (73.1%)	91 (26.9%)	2.02(1.31–3.11)*	1.77(1.04-3.00)*

* = Statistically significant

The model was adjusted for sex, age, marital status, educational status, occupation, household income, diagnosis date of hypertension, total comorbidities, clinical characteristics, physical activity level, tobacco consumption, alcohol consumption, khat consumption and frequency of EAFH. 

### Comparison of the nutritional status and dietary pattern between individuals with high and low consumption of foods away from home

Among individuals who eat away from home and among individuals with high frequency of eating away from home, 56.6% are overweight or obese (BMI ≥ 25). Similarly, among individuals with high frequency of EAFH, 74.9% had low fruit and vegetable consumption, while among those with low EAFH frequency, 56.5% had low fruit and vegetable consumption. This means that, among all people who eat away from home, a much higher proportion of those who eat frequently are overweight or obese and have low fruit and vegetable consumption compared to those who eat away from home less frequently (Table 8).

**Table 8:** Nutritional status and dietary patterns by frequency of eating away from home, Addis Ababa, January–March 2025

Variables	High EAFH frequency (%)	Low EAFH frequency (%)
BMI category		
Overweight or obese (BMI ≥ 25)	56.6	37.6
Fruit and vegetable consumption		
Low consumption	74.9	56.5

## Discussion

In this study, EAFH was measured using a composite index incorporating frequency, type, and reasons for consuming food prepared outside home. The prevalence of high EAFH frequency, defined as eating out three or more times per week, was 24.6% among hypertensive patients in Addis Ababa. A significant association was found between frequent EAFH and poor blood pressure control showing that individuals who ate away from home frequently had 2.02 times higher odds of having uncontrolled blood pressure compared to those who ate out less often.

EAFH in this study was less prevalent, with 17.4% eating fast food daily and 14.3% eating out three or more times per day. According to the CDC, between 2013 and 2016, about 36.6% of U.S adults consumed fast food on any day or single 24-hour period [28]. A study published in China reported that in 2017, the prevalence of eating out had increased to 55.6% among urban Chinese adults [29]. A national survey found that 11.3% of South Africans frequently consumed street food, while 6.8% regularly ate fast-food [30]. In Nigerian universities fast-food consumption is widespread, with 67% of students reporting daily consumption in one university [31]. Contrasting with these above prevalences this study shows less prevalence of EAFH.

The trend of eating away from home is increasing, especially in urban areas like Addis Ababa, and is associated with unhealthy dietary patterns and poor BP control [32]. In this study, high frequency of EAFH was associated with nearly double the odds of uncontrolled BP (AOR = 1.77, 95% CI: 1.04–3.00). When compared with international studies, the magnitude and direction of the association between EAFH and BP control of this study in Addis Ababa is consistent. For instance, a large cross-sectional study in rural China involving 29,611 adults found that eating away from home seven or more times per week was associated with a 67% higher risk of hypertension (OR = 1.67, 95% CI: 1.48–1.89), and this relationship showed a clear dose-response pattern [33]. Similarly, in Singapore, a study among university students reported that each additional meal eaten away from home per week increased the odds of prehypertension or hypertension by 5–6% (OR = 1.05, 95% CI: 1.01–1.09) [34]. These studies also noted that EAFH is linked to higher sodium intake, lower fruit and vegetable consumption and in the case of the Chinese study, that BMI mediated a significant portion of the relationship between EAFH and hypertension [35, 36].

This study shows that frequent consumption of meals outside the home is associated with a decline in nutritional status and a higher risk of obesity. This finding is consistent with evidences from both Ethiopia and other countries. A recent systematic review and meta-analysis found that the combined prevalence of overweight and obesity among Ethiopian adolescents was 10% (95% CI: 9–12%), with higher rates observed among students in private schools likely due to increased access to calorie-dense, processed foods and fast-food outlets [37].

In this study, blood pressure control was defined using the 2017 ACC/AHA guideline cutoff of < 130/80 mmHg. Despite this lower threshold, the control rate observed (31.1%) is comparable to findings from studies in Kenya (33.4%) [17] and the global PURE study (32.5%) [16], both of which used the conventional < 140/90 mmHg cutoff. In contrast, some systematic reviews from Ethiopia have reported higher control rates (around 50%) [19, 38], though these also applied the less strict threshold. The high prevalence of uncontrolled hypertension may indicate a need for more intensive monitoring, follow-up, and patient education by health care providers to improve treatment adherence and long-term control.

A 2024 systematic review and meta-analysis reported that the pooled prevalence of uncontrolled hypertension in Ethiopia was 51%. Specifically, in Addis Ababa, the prevalence of uncontrolled hypertension was even higher at 58%, corresponding to a BP control rate of approximately 42%. This figure is notably higher than our finding of 31.1% BP control [19]. Another systematic review and meta-analysis also reported the pooled prevalence of uncontrolled hypertension in Ethiopia to be 48%, suggesting a BP control rate of about 52%. This national estimate is higher than our finding [39]. This study is also comparable with other low-income African settings, where similarly low blood pressure control rates were reported in Kenya (33.4%), Cameroon (36.8%), and South Africa (42.0%) [35, 38].

Dietary habits showed high salt use (80.7%) and low fruit and vegetable intake (3.7% consumed fruit daily and 10.2% consumed vegetables daily). Similar trends of poor dietary habits have been observed in different areas of Ethiopia. For instance, a national study conducted revealed that only 1.5% of Ethiopians met the WHO’s recommendation of consuming five or more servings of fruits and vegetables daily, with rural residents having a slightly higher intake compared to those in urban areas [40]. Among hypertensive patients in Addis Ababa, 80.1% consumed inadequate fruits and vegetables, and this low intake was associated with increased cardiovascular risk [13]. Another study in Ethiopia also showed that among the participants 88% of them add salt to food at home during cooking [41].

This study shows that fruit and vegetable consumption is lower among individuals who frequently eat away from home. Although there is a lack of evidence about this in Ethiopian studies, it contrasts with some international findings, such as those from the United States, where research has indicated that vegetable consumption can be higher when eating out [42]. However, these findings are consistent with broader trends observed in low- and middle-income countries such as Burkina Faso, Ethiopia, Bangladesh, Tanzania, Nigeria, India, and Nepal. In these settings, eating away from home is relatively common especially among youth and migrants but the food consumed is often of poor nutritional quality, typically consisting of inexpensive, energy-dense staples that are filling yet lacking in essential nutrients such as fruits and vegetables. This mirrors the findings of the current study, where a lower intake of fruits and vegetables was associated with higher frequency of eating outside the home [43].

BMI analysis in this study revealed that 30.7% of participants were overweight and 12% were classified as obese. These results are consistent with other research conducted in the same setting and population. For instance, a 2023 study on adults in Addis Ababa reported that 29.7% of participants were overweight and 9% were obese, figures that closely resembles with these results [13]. Similarly, a 2018 study conducted in Addis Ababa public health facilities found that 36.7% of participants were overweight and 10.8% were obese [44]. A systematic review of over- nutrition among hypertensive patients in Southwest Ethiopia reported that 29% were overweight and 7% were obese, while the general adult population had a lower prevalence of 20.4% overweight and 5.4% obese [45].

BMI findings showed that 30.7% of participants were overweight and 12% obese, but BMI was not a significant predictor of BP control in this study. A study conducted in US indicated that, higher BMI is a consistent risk factor for hypertension and poor control [46], though the lack of association here may reflect the influence of other confounding factors.

These results show how important eating habits are for managing blood pressure and suggest that health programs should think about not just what people eat, but also where they eat and what exactly they consume. Even if majority of the participants thought EAFH affects BP control negatively, this knowledge doesn't necessarily translate into practice. This discrepancy suggests a potential disconnect between awareness and behavior, indicating a need to explore the underlying reasons why individuals, despite knowing better, still engage in dietary practices that negatively impact their blood pressure. A key area of uncertainty also lies in the source of excessive salt intake. It remains unclear whether the primary issue is uncontrolled salt usage in home-cooked meals or the consumption of high-sodium foods when eating away from home.

This study, as strength, has an institutional-based cross-sectional design that allows for a comprehensive assessment of dietary habits and blood pressure control within hypertensive patients. The use of standardized data collection tools enhances the reliability and validity of the findings. The study also addressed a largely unrecognized issue in Ethiopia by examining dietary practices and their impact on hypertension management. However, the cross-sectional design limits the ability to establish causal relationships between eating behaviors and blood pressure control. Self-reported data may be affected by recall and social desirability bias, and the sample may not fully represent all hypertensive patients in Addis Ababa as it was drawn from selected health institutions.

## Conclusion

A significant proportion of participants (68.9%) had uncontrolled blood pressure, indicating a critical public health challenge. The study identified a strong association between frequent EAFH and poor blood pressure control, with individuals who ate out three or more times per week being nearly twice as likely to have uncontrolled hypertension compared to those who rarely dined out. The results align with both global and national evidence linking EAFH to unhealthy dietary patterns, including high sodium intake and low consumption of fruits and vegetables, which are detrimental to hypertension management. The findings underscore the importance of raising awareness on EAFH risks, promoting home-cooked meals, enforcing nutritional guidelines, integrating dietary counseling and organizing community workshops to improve hypertension control.

## Supplementary information

Supplementary Material 1.

## Data Availability

A full data set and other materials about this study can be obtained from the corresponding author on reasonable request.

## Declarations

### Supplementary files

Supplementary Table S1–S8: Detailed tabulated data corresponding to the main tables (see Supplementary File).
