# Supplementary material for: Eating away from home among hypertensive patients in Addis Ababa: frequency and association with blood pressure control
Source: BMC Public Health. 2026 Mar 21;26:1499. doi: 10.1186/s12889-026-26871-x (PMC13151349; doi:10.1186/s12889-026-26871-x)
Supplement: Supplementary file 1 — Supplementary Material 1. [file 12889_2026_26871_MOESM1_ESM.docx]

**English version Questionnaire**

**Survey information**


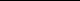

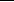
 Name of the hospital


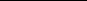
 Interviewer ID


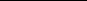
 Patient ID


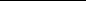
 Data completion date

1. **Demographic and socio-economic information**

| **No** | **Questions** | **Responses** | **Remark** |
| --- | --- | --- | --- |
| 101 | Sex | 1. Male  2. Female |  |
| 102 | What is your age? | **—————** in years |  |
| 103 | What is your marital status? | 1. Never married  2. Currently married  3. Divorced  4. Separated  5. Widowed |  |
| 104 | What is the highest level of school you have attended? | 1. Can’t read and write  2. Can read and write  3. Primary education (1-4)  4. Primary education (5-8)  5. Secondary education (9-12)  6. University or above  7. Technical or vocational |  |
| 105 | What is your occupation? | 1. Professional/technical/managerial  2. Sales and services  3. Skilled manual  4. Unskilled manual  5. Domestic service  6. Unemployed/Jobless  7. Other(specify) _____________ |  |
| 106 | How much is your monthly household income? | _____________ in ETB |  |
| 107 | How much is your monthly income? | _____________ in ETB |  |

1. **Clinical characteristics**

| **No** | **Questions** | **Responses** | **Remark** |
| --- | --- | --- | --- |
| 201 | When were you diagnosed with hypertension? | 1. Within the last 6 months 2. 6 months to 1 year ago 3. 1 to 3 years ago 4. More than 3 years ago 5. I don't remember |  |
| 202 | Do you have any of the following comorbid conditions or complications associated with hypertension?  (Select all that apply) | 1. Diabetes 2. Obesity 3. Heart disease (like coronary artery disease) 4. Chronic kidney disease 5. Stroke 6. No comorbidity 7. Other(specify) _____________ |  |
| 203 | If you have other chronic condition, do you take any medications for it? | 1. Yes 2. No |  |
| 204 | Which of the following best describes your current use of medication for hypertension? | 1. Currently taking medication for hypertension 2. Previously took medication for hypertension but not currently 3. Have been prescribed medication but have not started taking it 4. Have not been prescribed any medication for hypertension |  |
| 205 | Do you monitor your blood pressure at least twice a week? | 1. Yes 2. No |  |
| 206 | How often do you go to the health center or hospital for follow-up appointments for hypertension? | 1. Weekly 2. Monthly 3. Every 3 months 4. Every 6 months 5. Annually 6. As scheduled by the healthcare provider |  |

1. **Behavioral factors**

| **No** | **Questions** | | **Responses** | **Remark** |
| --- | --- | --- | --- | --- |
| **3.1 Physical activity** | | | | |
| Next, I am going to ask you about the time you spend doing different types of physical activity in a typical week. Please answer these questions even if you do not consider yourself to be a physically active person. Think first about the time you spend doing work. Think of work as the things that you have to do such as paid or unpaid work, study/training, household chores, seeking employment. In answering the following questions 'vigorous-intensity activities' are activities that require hard physical effort and cause large increases in breathing or heart rate, 'moderate-intensity activities' are activities that require moderate physical effort and cause small increases in breathing or heart rate. | | | | |
| **3.1.1 Work** | | | | |
| **3.1.1.1 Vigorous-intensity physical activities** | | | | |
| 301 | | Does your work involve vigorous-intensity activity (e.g., carrying heavy loads, construction work) for at least 10 minutes continuously? | 1. Yes 2. No | If No, go to 304 |
| 302 | | In a typical week, on how many days do you do vigorous-intensity activities as part of your work continuously? | Number of days: ___________ |  |
| 303 | | How much time do you spend doing vigorous-intensity activities at work on a typical day continuously? | Hours: ___________  Minutes: __________ |  |
| **3.1.1.2 Moderate-intensity physical activities** | | | | |
| 304 | | Does your work involve moderate-intensity activity (e.g., brisk walking, carrying light loads) for at least 10 minutes? | 1. Yes 2. No | If No, go to 307 |
| 305 | | In a typical week, on how many days do you do moderate-intensity activities as part of your work? | Number of days: ___________ |  |
| 306 | | How much time do you spend doing moderate-intensity activities at work on a typical day? | Hours: ___________  Minutes: __________ |  |
| **3.1.2 Travel to and from places** | | | | |
| The next questions exclude the physical activities at work that you have already mentioned. Now I would like to ask you about the usual way you travel to and from places. For example, to work, for shopping, to market, to place of worship. | | | | |
| 307 | | Do you walk or use a bicycle (pedal cycle) for at least 10 minutes continuously to get to and from places? | 1. Yes 2. No | If No, go to 310 |
| 308 | | In a typical week, on how many days do you walk or  bicycle for at least 10 minutes continuously to get to and from places? | Number of days: ___________ |  |
| 309 | | How much time do you spend walking or bicycling for  travel on a typical day? | Hours: __________  Minutes: ________ |  |
| **3.1.3 Recreational activities** | | | | |
| The next questions exclude the work and transport activities that you have already mentioned. Now I would like to ask you about sports, fitness and recreational activities (leisure) | | | | |
| 310 | | Do you do any vigorous-intensity sports, fitness or  recreational (leisure) activities that cause large  increases in breathing or heart rate like (running or football]) for at least 10 minutes continuously? | 1. Yes 2. No | If No, go to 313 |
| 311 | | In a typical week, on how many days do you do vigorous-intensity sports, fitness or recreational (leisure) activities? | Number of days: ___________ |  |
| 312 | | How much time do you spend doing vigorous-intensity sports, fitness or recreational activities on a typical day? | Hours: ___________  Minutes: __________ |  |
| 313 | | Do you do any moderate-intensity sports, fitness or recreational (leisure) activities that cause a small increase in breathing or heart rate such as brisk walking, (cycling, swimming and volley ball) for at least 10 minutes continuously? | 1. Yes 2. No | If No, go to 316 |
| 314 | | In a typical week, on how many days do you do moderate-intensity sports, fitness or recreational (leisure) activities? | Number of days: ___________ |  |
| 315 | | How much time do you spend doing moderate-intensity  sports, fitness or recreational (leisure) activities on a  typical day? | Hours: ___________  Minutes: __________ |  |
| **3.1.4 Sedentary behavior** | | | | |
| The following question is about sitting or reclining at work, at home, getting to and from places, or with friends including time spent sitting at a desk, sitting with friends, traveling in car, bus, train, reading, playing cards or watching television, but do not include time spent sleeping. | | | | |
| 316 | | How much time do you usually spend sitting or reclining on a typical day? | Hours: ___________  Minutes: __________ |  |
| **3.2 Tobacco consumption** | | | | |
| Now I am going to ask you some questions about tobacco consumption | | | | |
| 317 | | Do you currently smoke any tobacco products, such as cigarettes, cigars or pipes? | 1. Yes 2. No | If No, go to 322 |
| 318 | | Do you currently smoke tobacco products daily? | 1. Yes 2. No   Yes  No | If No, go to 322 |
| 319 | | How old were you when you first started smoking? | 1. ————— in years 2. Don’t know / Not sure |  |
| 320 | | Do you remember how long ago it was? | ————— in years  ————— in months  ————— in weeks | If known, go to 321(1) |
| 321 | | On average, how many of the following do you smoke  each day? | 1. Manufactured cigarettes 2. Hand-rolled cigarettes 3. Pipes full of tobacco 4. Cigars, cheroots, cigarillos 5. Other (specify): _________________ |  |
| **3.3 Alcohol consumption** | | | | |
| Now I am going to ask you some questions about alcohol consumption | | | | |
| 322 | | Have you ever consumed an alcoholic drink such as beer, wine, spirits, fermented cider, Tej, Tella, Areke? | 1. Yes 2. No | If No, go to 330 |
| 323 | | Have you consumed an alcoholic drink within the past 12 months? | 1. Yes 2. No | If No, go to 330 |
| 324 | | During the past 12 months, how frequently have you  had at least one alcoholic drink? | 1. Daily 2. 5-6 days per week 3. 1-4 days per week 4. 1-3 days per month 5. Less than once a month |  |
| 325 | | Have you consumed an alcoholic drink within the past 30 days? | 1. Yes 2. No | If no, go to 330 |
| 326 | | During the past 30 days, on how many occasions did you have at least one alcoholic drink? | 1. ————— in number 2. Don’t know / Not sure |  |
| 327 | | During the past 30 days, when you drank alcohol, on average, how many standard alcoholic drinks did you have during one drinking occasion? | 1. ————— in number 2. Don’t know / Not sure |  |
| 328 | | During the past 30 days, what was the largest number of standard alcoholic drinks you had on a single occasion, counting all types of alcoholic drinks together? | 1. Largest number ————— 2. Don’t know / Not sure |  |
| 329 | | During the past 30 days, how many times did you have for men: five or more for women: four or more standard alcoholic drinks in a single drinking occasion? | 1. Number of times ————— 2. Don’t know / Not sure |  |
| **3.4 Khat consumption** | | | | |
| Now I am going to ask you some questions about khat consumption | | | | |
| 330 | | Do you use Khat? | 1. Yes 2. No | If no, go to 401 |
| 331 | | If yes, how frequent do you chew khat? | 1. Daily 2. Three times a week 3. Once a week 4. Once a month 5. Don’t remember |  |
| 332 | | What is the amount of khat you chew per session? | 1. Mild 2. Moderate 3. Heavy 4. Don’t remember |  |

1. **Dietary factors**

| **No** | **Questions** | **Responses** | **Remark** |
| --- | --- | --- | --- |
| **4.1 Salt restriction** | | | |
| 401 | How frequently do you use salt at your home while cooking? | 1. Never 2. Rarely 3. Sometimes 4. Often 5. Always |  |
| 402 | Do you regularly limit your salt intake? | 1. Yes 2. No |  |
| 403 | What methods do you use to reduce salt in your diet? (Select all that apply) | 1. Avoid processed foods 2. Add less salt during cooking 3. Avoid salty snacks 4. Other (specify) _________________ |  |
| **4.2 Fruit and vegetable consumption** | | | |
| 404 | Over the past week or 7 days, on how many days did you eat fruit? | 1. Number of days ————— 2. Don’t know / Not sure |  |
| 405 | Over the past week or 7 days, on how many days did you eat vegetables? | 1. Number of days ————— 2. Don’t know / Not sure |  |
| 406 | Over the past week or 7 days, how many servings of each type of fruit do you eat at each occasion? | 1. Apples:  - Small (1 small apple = 1 serving) - Medium (1 medium apple = 1 servings) - Large (1 large apple = 1.5-2 servings) - Servings: _______  1. Bananas:  - Small (1 small banana = 0.75 serving) - Medium (1 medium banana = 1 serving) - Large (1 large banana = 1.5 serving) - Servings: _______  1. Oranges:  - Small (1 small orange = 0.75 serving) - Medium (1 medium orange = 1 serving) - Large (1 large orange = 1.5 serving) - Servings: _______  1. Berries:  - Small (1/2 cup of berries = 0.5 serving) - Medium (1 cup of berries = 1 serving) - Large (1.5 cups of berries = 1.5 servings) - Servings: _______  1. Grapes  - Small (½ cup of grapes) = 0.5 servings - Medium (1 cup of grapes) = 1 serving - Large (1.5 cups of grapes) = 1.5 servings - Servings: _______  1. Mangoes:  - Small (1 small mango) = 1 serving - Medium (1 medium mango) = 1.5–2 servings - Large (1 large mango) = 2.5–3 servings - Servings: ________ |  |
| 407 | Over the past week or 7 days, how many servings of each type of vegetables do you eat at each occasion? | 1. Spinach (Cooked):   - Small (½ cup) = 0.5 servings - Medium (1 cup) = 1 serving - Large (1.5 cups) = 1.5 servings - Servings: _______   2. Carrots:   - Small (1 small carrot) = 0.5 servings - Medium (1 medium carrot) = 1 serving - Large (1 large carrot) = 1.5 serving - Servings: _______  1. Broccoli (Cooked):  - Small (½ cup) = 0.5 servings - Medium (1 cup) = 1 serving - Large (1.5 cups) = 1.5 servings - Servings: _______   4. Tomatoes:   - Small (½ medium tomato) = 0.5 servings - Medium (1 medium tomato) = 1 serving - Large (1.5 medium tomatoes) = 1.5 servings - Servings: _______   5. Peppers:   - Small (½ medium pepper) = 0.5 serving - Medium (1 medium pepper) = 1 serving - Large (1.5 medium peppers) = 1.5 servings - Servings: _______   6. Onions:   - Small (½ medium onion) = 0.5 serving - Medium (1 medium onion) = 1 serving - Large (1.5 medium onions) = 1.5 serving - Servings: _______ |  |

1. **Eating away from home**

| **No** | **Questions** | **Responses** | **Remark** |
| --- | --- | --- | --- |
| 501 | Over the past 30 days, how many times did you buy food at a fast-food restaurant (Chips, sandwiches, doughnuts, pasty, burger, pizza, fried foods and ice cream)? | 1. Never or rarely 2. 1 time per month 3. 2-3 times per month 4. 1-2 times per week 5. 3-4 times per week 6. 5-6 times per week 7. 1 time per day 8. 2 times per day 9. 3 or more times per day |  |
| 502 | Not including the fast-food restaurants listed above, in the past 30 days, how many times did you  buy food at any other sit down (full service) restaurant and order from a waiter/waitress? | 1. Never or rarely 2. 1 time per month 3. 2-3 times per month 4. 1-2 times per week 5. 3-4 times per week 6. 5-6 times per week 7. 1 time per day 8. 2 times per day 9. 3 or more times per day |  |
| 503 | Over the past 30 days, how many times did you buy food from an all-you-can-eat buffet, such as college or university dining halls, hotels or resorts? | 1. Never or rarely 2. 1 time per month 3. 2-3 times per month 4. 1-2 times per week 5. 3-4 times per week 6. 5-6 times per week 7. 1 time per day 8. 2 times per day 9. 3 or more times per day |  |
| 504 | Over the past 30 days, how many times did you consume sweet foods that are prepared away from home (E.g. sugar, honey, chocolates, candies, cookies and cakes)? | 1. Never or rarely 2. 1 time per month 3. 2-3 times per month 4. 1-2 times per week 5. 3-4 times per week 6. 5-6 times per week 7. 1 time per day 8. 2 times per day 9. 3 or more times per day |  |
| 505 | In the last week (7 days), how many meals prepared away from home do you consume (excluding snacks)? | Breakfast _________ days per week  Lunch _________ days per week  Dinner _________ days per week |  |
| 506 | In the last week (7 days), How often do you consume takeaway food? | 1. Never or rarely 2. 1 time per month 3. 2-3 times per month 4. 1-2 times per week 5. 3-4 times per week 6. 5-6 times per week 7. 1 time per day 8. 2 times per day 9. 3 or more times per day |  |
| 507 | Which types of takeaway meals do you usually consume? | 1. Fast-food such as Burgers, fries, chicken nuggets, pizza 2. Casual dining meals like international cuisines 3. Street foods like Injera, kokor or pasti, bonbolino and ertib 4. Cakes or bakeries 5. Sweets and snacks |  |
| 508 | Which types of meals do you usually consume outside your home? | 1. Fast-food such as Burgers, fries, chicken nuggets, pizza 2. Casual dining meals like international cuisines 3. Street foods like, Injera kokor or pasti, bonbolino and ertib 4. Cakes or bakeries 5. Sweets and snacks |  |
| 509 | Why do you eat away from home? Select all that apply) | 1. Convenience 2. For socializing purpose 3. Peer influence 4. For luxury 5. Lack of cooking skills 6. Social media marketing 7. Taste preference 8. Busy lifestyle 9. Other (Specify)_____________ |  |
| **Knowledge and attitude questions** | | | |
| 510 | Are you aware of the dietary recommendations for managing hypertension? (e.g. low salt, more fruits/vegetables intake) | 1. Yes 2. No |  |
| 511 | Do you believe that eating away from home affects your blood pressure control? | 1. Yes 2. No |  |
| 512 | If yes, how do you think it affects it? | 1. Positively 2. Negatively |  |

1. **Measurements**

| **No** | **Questions** | **Readings** | **Remark** |
| --- | --- | --- | --- |
| **6.1 Blood pressure measurement** | | | |
| 601 | Can I measure your blood pressure? |  |  |
| **6.2 Anthropometric measurement** | | | |
| 602 | Height (in centimeter) | ________________________ |  |
| 603 | Weight (in kilogram) | ________________________ |  |
